# Effect of Prenatal Iron Supplementation Adapted to Hemoglobin Levels in Early Pregnancy on Fetal and Neonatal Growth—ECLIPSES Study

**DOI:** 10.3390/nu16030437

**Published:** 2024-02-01

**Authors:** Sandra Díaz-Torres, Andrés Díaz-López, Victoria Arija

**Affiliations:** 1 Nutrition and Mental Health (NUTRISAM) Research Group, Universitat Rovira i Virgili (URV), 43204 Reus, Spain; sandraditorres@gmail.com (S.D.-T.); andres.diaz@urv.cat (A.D.-L.); 2 Institut d’Investigació Sanitària Pere Virgili (IISPV), 43005 Tarragona, Spain; 3 Collaborative Group on Lifestyles, Nutrition, and Tobacco (CENIT), Tarragona-Reus Research Support Unit, Jordi Gol Primary Care Research Institute, 43202 Reus, Spain

**Keywords:** iron supplementation, pregnancy, fetal development, fetal biometrics, ECLIPSES study

## Abstract

In this randomized clinical trial, we evaluated the effects of prenatal iron supplementation adapted to pregnant women’s initial hemoglobin (Hb) levels on fetal growth parameters until birth in women from the Mediterranean coast of northern Spain. All (*n* = 791) women were iron-supplemented during pregnancy according to Hb levels at the 12th gestational week: stratum 1 (Hb: 110–130 g/L) received 40 or 80 mg iron daily; stratum 2 (Hb > 130 g/L) received 40 or 20 mg iron daily. Fetal biometric and anthropometric measurements were evaluated in the three trimesters and at birth, respectively. In stratum 1, using 80 mg/d instead of 40 mg/d increased the risk of fetal head circumference > 90th percentile (OR = 2.49, *p* = 0.015) at the second trimester and fetal weight (OR = 2.36, *p* = 0.011) and femur length (OR = 2.50, *p* = 0.018) < 10th percentile at the third trimester. For stratum 2, using 40 mg/d instead of 20 mg/d increased the risk of fetal head circumference > 90th percentile (OR = 3.19, *p* = 0.039) at the third trimester. A higher risk of delivering an LGA baby (OR = 2.35, *p* = 0.015) for birthweight was also observed in stratum 1 women receiving 80 mg/d. It is crucial to adjust the prenatal iron supplementation to each pregnant woman’s needs, i.e., adapted to their initial Hb levels, to achieve optimal fetal development, since excessive iron doses appear to adversely influence fetal growth.

## 1. Introduction

Iron deficiency (ID), defined as serum ferritin (SF) < 15 µg/L according to the WHO, is the most common nutritional deficiency worldwide and disproportionately affects pregnant women, being the main cause of anemia (generally defined as a hemoglobin (Hb) value < 110 g/L) in pregnancy [1]. Although the percentage of pregnant women with iron deficiency without anemia is unknown, we do know that the global prevalence of anemia in pregnant women is approximately 36%, with an average of 17.2% in developed countries and 42.6% in developing countries [2,3]. Therefore, this condition can be considered a significant public health concern.

During pregnancy, iron requirements increase considerably to meet the demands of the fetoplacental unit, the increase in maternal blood volume, and to compensate for iron loss at delivery [4]. Therefore, pregnant women with low iron reserves are not properly prepared to face the increased requirements. Moreover, this cannot always be resolved through the conventional dietary intake of iron-containing foods [5], which are often below nutritional needs, heightening the risk of iron deficiency anemia. Conversely, in women who are already iron-replete and/or have the genetic alteration in the HFE gene of hereditary hemochromatosis, supplementing their diet with iron can lead to iron overload [6], increasing the risk of certain pregnancy complications.

Iron is an essential micronutrient. It has a pivotal influence on the in-uterus environment, and therefore on fetal growth and development, as it influences physiological processes such as oxygen transport and storage, cellular energy metabolism, DNA biosynthesis, the synthesis of collagen and neurotransmitters, the immune function, and brain development [4]. An unbalanced iron status during pregnancy, whether due to deficiency or overload, increases the risk of preterm birth and of having a low birthweight (LBW)/intrauterine growth-restricted baby [7]. It also increases the risk of having a high birth weight or large-for-gestational-age (LGA) baby due to inflammatory mechanisms and oxidative stress related to iron overload [8]. These factors could lead to alterations in maternal–fetal glycemic control [8], particularly when women who are already replete with iron supplement their dietary iron intakes. However, current evidence in this field is somewhat contradictory and merits further convincing evidence from randomized controlled trials. Given that iron appears to show a U-shaped risk curve [7], ensuring optimal prenatal iron status should be a major public health priority to avoid negative consequences for both the mother and the baby.

Iron supplementation during pregnancy is a widely used strategy for preventing ID anemia (determined according to Hb < 110 g/L and SF < 15 µg/L). However, the optimal dose and the ideal time for supplementation, particularly for non-anemic pregnant women, remain controversial topics [7]. Currently, the WHO recommends daily supplementation with 30–60 mg of elemental Fe/d throughout pregnancy. In settings where the prevalence of anemia in pregnant women is 40% or more, a daily dose of 60 mg recommended [9]. These recommendations are based on a Cochrane review published in 2012 and updated in 2015, which points out that pregnant women supplemented daily with iron are less likely to have a baby with LBW [10,11].

A 2023 meta-analysis [12] investigating the impact of prenatal iron supplementation on pregnancy outcomes suggests that daily oral iron supplements, in comparison to a placebo, may reduce the occurrence of iron deficiency anemia at term and the incidence of LBW newborns. Additionally, it is likely to decrease the incidence of small-for-gestational-age (SGA) newborns. In this meta-analysis, eight randomized controlled trials (RCTs) involving 2822 participants were conducted across various locations, including Italy [13], Sweden [14], Finland [15], Iran [16,17,18,19], the United States [20], and China [21]. In all of the included trials, interventions were initiated at no later than 20 weeks of gestation with the total daily dose of elemental iron ranging from 30 to 200 mg.

However, a review conducted in in 2017 [22], considering various RCTs and observational studies, found that iron intake of supplements among pregnant women with a low risk of ID may have adverse effects on birth outcomes. This includes an increased likelihood of giving birth to an SGA baby and having hypertension disorder [23], as well as a shorter duration of gestation and a higher risk of LBW [24]. In addition, other evidence has also suggested that maternal iron supplementation could lead to an increased size of the baby and adiposity at birth in terms of weight, head circumference, body mass index, and various skinfold thicknesses [25]. However, previous studies have mainly focused on birth outcomes, and relatively little is known about the implications of prenatal iron supplementation on fetal growth based on fetal ultrasound measurements, which provide reliable insights into intrauterine growth. In this regard, a prospective observational study conducted in Republic of Korea in 2013 [26] with 1563 pregnant women found that babies of mothers with a higher iron intake from their diet and supplements (>17.04 mg/d), had, at the middle of pregnancy, a smaller biparietal diameter, abdominal circumference, and femur length by 0.41 cm, 0.41 cm, and 0.07 cm, respectively. Although the fetal biometry was only assessed with ultrasonography at a single timepoint (mid-pregnancy), this suggests that excessive exposure to maternal iron in utero could have harmful effects on fetal growth. Therefore, routine iron supplementation in non-anemic women is not advisable as it could be potentially harmful [23].

In this context, our research group [27], as well as other researchers [28,29], has emphasized the importance of individualizing iron supplementation during pregnancy. Although it proves beneficial for maternal biochemical iron markers and the child’s birth weight in women with low iron stores before pregnancy, these effects are less evident in women starting pregnancy with sufficient iron levels, i.e., SF ≥ 20 μg/L [27].

Optimal fetal growth is a powerful indicator of both intrauterine and postnatal health status [30]. Neonates classified as SGA, LBW, LGA, or macrosomia have an elevated risk of perinatal complications, including obstetric trauma, fetal death, respiratory distress syndrome, hypoglycemia, and hypothermia. In addition, they are more predisposed to cardiometabolic disorders, including obesity, type 2 diabetes, and hypertension [31], as well as experiencing difficulties in cognitive, motor, and emotional development throughout life [32]. Therefore, it is crucial to diagnose alterations in fetal growth early to implement the necessary interventions, such as changing maternal risk factors, and, in extreme cases, planning birth before term (induction or cesarean section) to prevent complications [33]. However, to our knowledge, there are no RCTs that consider fetal biometrics throughout pregnancy and focus on the effect of iron supplementation on fetal growth. Therefore, further research is needed. The present trial aimed to determine the effects of prenatal iron supplementation adapted to Hb levels in early pregnancy on fetal growth until birth in non-anemic pregnant women from the Mediterranean coast of northern Spain. We hypothesized that prenatal iron supplementation adapted to initial Hb levels in non-anemic women (i.e., in women with normal Hb values of between 110 and 130 g/L, a daily dose of 40 mg of iron as opposed to 80 mg, and in women with normal–high Hb values of >130 g/L, a daily dose of 20 mg of iron compared to 40 mg) could be the best option for improving fetal and newborn growth. 

## 2. Materials and Methods

### 2.1. Study Design and Participants

The present investigation is part of the ECLIPSES (Ensayo CLInico Para Suplementar con hierro a EmbarazadaS) study [34], a community-randomized controlled trial involving 791 pregnant women recruited by midwives at their primary care centers in the province of Tarragona (Catalonia, Spain) between 2013 and 2017. A recruitment visit was performed before the 12th week of gestation. During this visit, Hb levels were checked to ensure that pregnant women met the study inclusion criteria of non-anemia, and to establish their initial Hb levels without an upper limit on concentration. Subsequently, there were three interviews with the women during the pregnancy (in the 12th, 24th, and 36th weeks) and one interview after delivery. Furthermore, women attended routine pregnancy visits with their midwives and obstetricians. Eligible participants were healthy adult women over 18 years old, with a gestation time ≤ 12 weeks, without laboratory values compatible with anemia (Hb ≥ 110 g/L at week 12), understanding at least one of the two official languages of the State (Spanish or Catalan), and the ability to understand the characteristics of the study. The exclusion criteria were as follows: multiple pregnancy, taking > 10 mg of daily iron supplements during the three months prior to the 12th week of gestation, hypersensitivity to egg protein, and previous severe or chronic disease that could affect their nutritional status (immunosuppression, cancer, diabetes, malabsorption, or liver disease). This trial was registered at ClinicalTrials.gov (identifier: NCT03196882) and the EU Clinical Trials Register (identifier: EUCTR-2012-005480-28). Ethical approval was given by Committee of the Pere Virgili Institute for Health Research (IISPV) and all participants provided written informed consent. The trial complies with the tenets of the Declaration of Helsinki. 

Participants were allocated to two different active interventions (called strata) depending to their baseline Hb levels, as follows: (1) stratum 1: pregnant women with Hb values of 110–130 g/L, which we call normal Hb, were randomly assigned to receive a daily dose of 40 or 80 mg of iron; and (2) stratum 2: women with Hb levels of >130 g/L, called medium-high, were randomly assigned to receive 40 or 20 mg of daily iron supplements. The doses of 20 mg, 40 mg, and 80 mg per day of elemental iron correspond to 150 mg, 300 mg, and 600 mg of ferrimanitol ovoalbumin. Midwives distributed the iron supplements to each woman according to the stratum. From week 12 onwards and continuing up to partum, women were advised to take one tablet per day until their next visit, at which time they should return any remaining tablets to assess compliance to the intervention. An independent researcher compared the number of pills left over. Compliance was considered “good” when women remembered to take the supplement five or more days per week. The intervention was triple-blinded, so that participants, healthcare providers, and researchers did not know which iron prescription each woman received until the end of the study. The ECLIPSES protocol is detailed elsewhere [34]. 

### 2.2. Outcomes Measurements

Fetal growth was measured repeatedly by ultrasound scan based on the recommendations of the International Society of Ultrasound in Obstetrics and Gynecology. All women who participated in the trial had an ultrasound scan during prenatal care visits, at the 1st (mean (SD): 13.0 (1.0) weeks of gestation; 81.2% were collected at or prior to 13th week), 2nd (23.3 (2.3) weeks of gestation), and 3rd (37.8 (1.8) weeks of gestation) trimesters to obtain fetal biometry from medical records. Fetal biometric measurements (in mm) were recorded longitudinally including crown–rump length (CRL) and biparietal diameter (BD) in the 1st trimester, and BD, head circumference (HC), abdominal circumference (AC), and femur length (FL) in the 2nd and 3rd trimesters. Estimated fetal weight (EFW) was calculated using Hadlock’s formula [35], based on HC, AC, and FL. All fetal growth parameter values were corrected for gestational age (weeks) at the time of ultrasound measurement using the residual method [36]. Gestational age was determined by the reported last menstrual period at recruitment, or by ultrasound estimation if it disagreed within 7 days. The sex of the children was also recorded.

Each fetal biometry measurement (BD, HC, CA, FL, and EFW) was analyzed as a continuous variable. To classify the group at higher risk, fetuses with lower and higher growth than expected at the second and third trimesters were also defined as a gestational age-adjusted EFW, FL, HC, BD, or AC below the 10th percentile and above the 90th percentile [37] in the study cohort, respectively. Fetuses with growth between the 10th and 90th percentile were considered to be appropriate for gestational age and were used as the reference group.

Birth weight (g), length (cm), and HC (cm) were measured just after birth by qualified and trained obstetricians or midwifery nurses in newborn anthropometry using standard procedures. Small-for-gestational age (SGA) and large-for-gestational age (LGA) were defined as a gestational age- and sex-specified birthweight, length, or HC below the 10th percentile and above the 90th percentile according to the INTERGROWTH-21st standards as reference, respectively [30].

### 2.3. Assessment of Covariates

At enrolment and once in each trimester of pregnancy, women were interviewed by the midwives and the study researchers during their visits to the ASSIR centers. Information on maternal age, socioeconomic level, educational level, and lifestyle habits, as well as their medical and obstetric history (including parity (primiparous vs. multiparous), planned pregnancy (yes vs. no)) were collected in individual interviews, as has also been reported previously [34]. Family socioeconomic status (SES) was calculated by combining information on occupational status (classified according to the Catalan classification of occupations, CCO-2011 [38]) and educational level, then categorized as low, middle, or high. Women’s educational levels were categorized as low (primary school or less), medium (secondary studies), or high (university studies or more). The Fagerström questionnaire [39] was used to assess smoking, and women were classified as current, former, and never smokers. The International Physical Activity Questionnaire (IPAQ) [40] was used to record the physical activity (PA) of the participants. PA was assessed by calculating the total metabolic equivalents (METs) based on frequency and duration of walking and moderate- and vigorous-intensity activities and classified as sedentary/low (<600 METs min/week), moderate (≥600–1200 METs min/week), or high (≥1200 METs min/week). Eating habits during pregnancy were evaluated using a self-administered food frequency questionnaire covering 45 food groups validated in our population. Based on this, we used the official French food composition table (REGAL—Répertoire Général des Aliments) [41] to extract daily energy intake (kcal), macronutrients and micronutrients (including iron (mg), vitamin C (mg), and fiber intake (g)), which was complemented by the Spanish food composition table [42]. Alcohol consumption was assessed as yes or no. Maternal weight (in kg to the nearest 0.1 kg) and height (in cm to the nearest 0.1 cm) were also measured (calibrated SECA^®^ brand scale with stadiometer) in early pregnancy and during follow-up, and BMI (in kg/m^2^) was calculated. Based on the criteria proposed by the WHO, women were classified as normal weight (BMI 18.5–24.9 kg/m^2^), overweight (BMI 25.0–29.9 kg/m^2^), or obese (BMI ≥ 30kg/m^2^) [43]. Gestational weight gain (GWG) was calculated as the difference between the weights measured at the 1st and 3er trimester visits.

In each trimester of pregnancy, blood samples were collected for biochemical determinations (Hb and serum ferritin and cortisol) and for DNA extraction (HFE gene). Hb values were immediately measured using a Coulter Gen-S analyzer (Coulter, Hialeah, FL, USA). Plasma was processed and stored at −80 °C at the Biobank of the IISPV (Tarragona) until analysis. This allowed for analyzing all samples together using the same kit, minimizing potential bias. SF was determined via turbidimetric immunoassay, as described previously [44]. Cortisol was assessed by a competitive immunoassay that uses direct chemiluminescence, using the systems ADVIA Centaur^®^, ADVIA Centaur XP, and ADVIA Centaur XPT. Iron deficiency anemia was defined as serum ferritin (SF) < 15 μg/L. Genetic determinations of HFE gene mutations (C282Y, H63D, and S65C) were performed using DNA extracted from leukocytes with the polymerase chain reaction technique.

### 2.4. Statistical Analysis

Statistical analyses were performed according to the intention to treat (ITT) principal of treatment allocation, considering all information on fetal biometry in each trimester and neonatal birth anthropometries available from medical records. Descriptive statistics were used to characterize the population of pregnant women by iron supplementation groups in stratum 1 and stratum 2. Quantitative variables are presented as mean ± SD, while categorical variables as a number (%). Between-group comparisons within each stratum were conducted using either Student’s T-test or the chi-square test, as deemed appropriate.

To assess the effect of different doses of prenatal iron supplementation (stratum 1: 40 mg/d (reference) vs. 80 mg/d; stratum 2: 20 mg/d (reference) vs. 40 mg/d) on fetal growth characteristics (EFW, FL, HC, BD, and AC) in the second and third trimester of pregnancy, and at birth (birth weight, length, and HC) were used multivariable linear regression models. Confounding variables in our analyses were primarily determined a priori and based on our knowledge and earlier literature. Maternal covariates included age (years), early-pregnancy BMI categories (normal weight (reference), overweight, obese), social class (low (reference), medium, high), smoking during pregnancy (no (reference), yes), fiber intake in the first trimester, log-transformed serum ferritin (µg/L) in the first trimester, cortisol (µg/dl) in the first trimester, and sex of baby. All third trimester and birth analyses were also adjusted for gestational weight gain (kg). The results were expressed as β-coefficients with their 95% confidence interval (CI).

We also assessed each fetal growth parameter as a binary outcome. Separate multivariate-adjusted logistic regression analyses, with a similar approach for selecting confounders, were applied to estimate the odds ratio (OR and 95% CI) of low (<10th percentile vs. 10th to 90th percentile (reference)) and high (>90th percentile vs. 10th to 90th percentile (reference) in the study cohort) fetal growth for gestational age-adjusted EFW, FL, HC, BD, and AC in the second and third trimesters according to different iron supplementation doses. Similarly, multivariable logistic regression models were also performed to assess the risks of SGA and LGA for each neonatal anthropometric measurement examined at birth (i.e., birth weight, length, and HC) separately. *P*-value below 0.05 was considered statistically significant. Statistical analyses were performed using STATA version 15.0 (Stata Corp LP, College Station, TX, USA).

## 3. Results

### 3.1. Characteristics of the Study Participants

**Figure 1:**
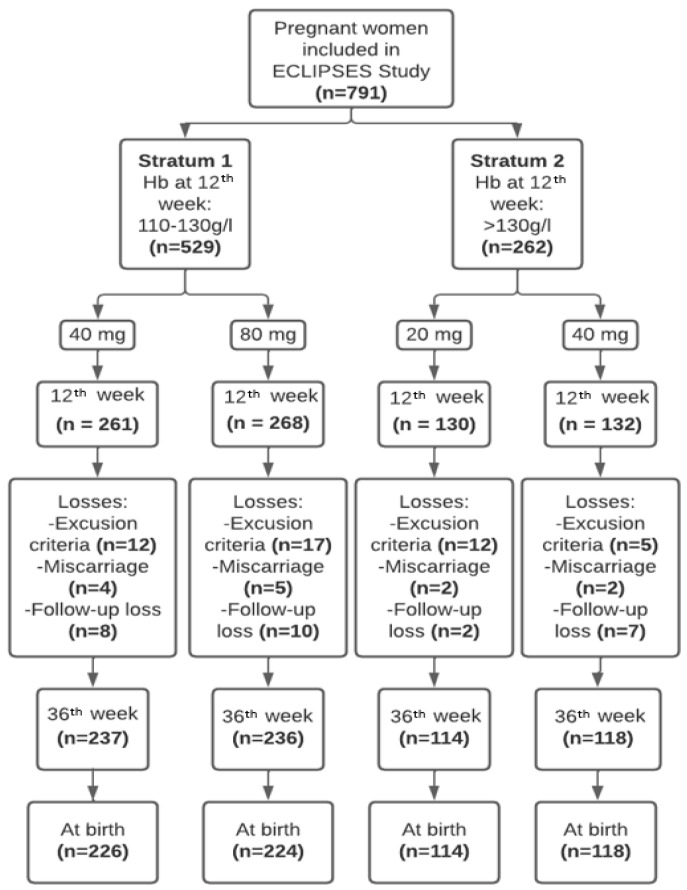
Flowchart of this study.

Figure 1 presents the flowchart of the sample study. The baseline characteristics of the women according to iron supplementation are reported in Table 1. There were no statistically significant differences within each stratum in the sociodemographic, lifestyle, or iron status characteristics. We observed a high compliance to the intervention throughout pregnancy, around 94%.

### 3.2. Effect of Iron Supplementation, Adjusted to the Pregnant Women’s Initial Individual Iron Status, on Mean Growth Values of the Fetus and at Birth

Supplemental Table S1 shows the comparison of fetal biometric measurements, including EFW, FL, HC, BD, and AC in the second and third trimesters of pregnancy, and the newborns’ anthropometric measurements, such as birth weight, length, and HC between iron supplementation groups of each stratum. There are no differences between them. After adjusting for possible confounders (Table 2), no statistically significant differences were found in the mean growth values between the groups within each stratum.

### 3.3. Effect of Iron Supplementation, Adjusted to the Pregnant Women’s Initial Individual Iron Status, on Optimal Fetal Growth (between the 10th and 90th Percentile)

**Figure 2:**
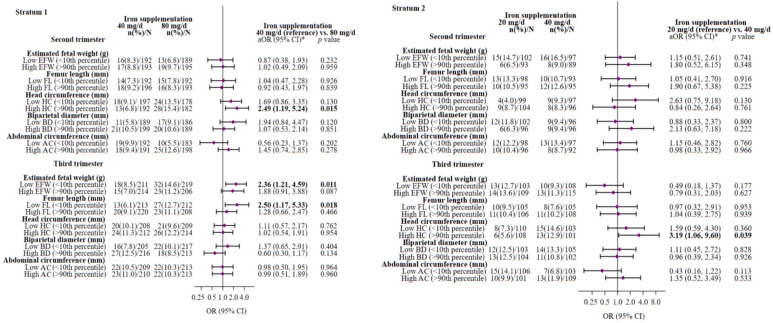
Multivariable-adjusted ORs (95%(CIs)) of low (<10th percentile) and high (>90th percentile) fetal growth in the second and third trimester pregnancy associated with maternal iron supplementation throughout pregnancy on stratum 1 (80 or 40 mg/d) and stratum 2 (40 or 20 mg/d). The reference group was 40 mg/d dose group for stratum 1 and 20 mg/d dose group for stratum 2. Abbreviations: EFW—estimated fetal weight; FL—femur length; HC—head circumference; BD—biparietal diameter; AC—abdominal circumference. All fetal growth parameter values were corrected for gestational age (weeks) at the time of ultrasound measurement using residual method. * Models were adjusted for sex of baby, maternal age (years), maternal early-pregnancy BMI categories (normal weight (ref.), overweight, obese), family SES (low (ref.), medium, high), smoking during pregnancy (no (ref.), yes), fiber intake at the first trimester, log-serum ferritin (µg/L) at the first trimester, cortisol (µg/dl) at the first trimester, carrier of HFE gene mutation (no (ref.), yes for stratum 2), and gestational weight gain (kg, only for third trimester analysis). The significance of numbers in bold is *p*-value < 0.05. Low (<10th percentile) or high (>90th percentile) fetal growth versus normal fetal growth (10th to 90th percentile) for gestational age-adjusted EFW, FL, HC, BD, or AC based on distribution percentile from the study population as reference. The diamonds represent OR and the whisker plots represent 95% CIs. n—number of cases infants (%); N—number of total infants.

Figure 2 presents the adjusted multivariable odds ratios (ORs) and their 95% confidence intervals (CIs) for low fetal growth (<10th percentile) and high fetal growth (>90th percentile) in the second and third trimesters of pregnancy between iron supplementation groups. Adjusting for possible confounding factors, we found that a daily iron supplementation of 80 mg, as opposed to 40 mg, during pregnancy (stratum 1) increased the risk of having fetuses with a HC above the 90th percentile (OR = 2.49; 95% CI 1.19–5.24; *p* = 0.015) in the second trimester, and having fetuses with an EFW (OR = 2.36; 95% CI 1.21–4.59; *p* = 0.011) and FL (OR = 2.50; 95% CI 1.17–5.33; *p* = 0.018) below the 10th percentile in the third trimester. It showed a non-significant trend towards a higher risk of having fetuses of high EFW (>90th percentile) in this last trimester (OR = 1.88; 95% CI 0.91–3.88; *p* = 0.087). In stratum 2, taking 40 g/d of iron prenatally instead of 20 mg/d increased the risk of having fetuses with a HC above the 90th percentile (OR = 3.19; 95% CI 1.06–9.60; *p* = 0.039) in the third trimester.

As a sensitivity analysis to assess the robustness of our findings, we replicated the main analysis defining low and high fetal growth in the second and third trimester as <25th percentile and >85th percentile, respectively. The results indicated consistent trends (Supplemental Figure S1).

### 3.4. Effect of Iron Supplementation, Adjusted to the Pregnant Women’s Initial Individual Iron Status, on the Risk of SGA or LGA at Birth

**Figure 3:**
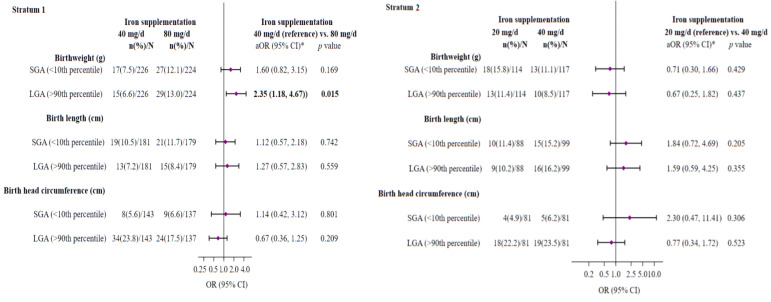
Multivariate-adjusted ORs (95%(CIs)) of SGA or LGA infant for neonatal anthropometric measurements at birth associated with maternal iron supplementation throughout pregnancy on stratum 1 (40 or 80 mg/d) and stratum 2 (40 or 20 mg/d). The reference group was 40 mg/d dose group for stratum 1 and 20 mg/d dose group for stratum 2. Abbreviations: SGA—small for gestational age; LGA—large for gestational age. * Models were adjusted for sex of baby, maternal age (years), maternal early-pregnancy BMI categories (normal weight (ref.), overweight, obese), family SES (low (ref.), medium, high), smoking during pregnancy (no (ref.), yes), fiber intake at the first trimester, log-serum ferritin (µg/L) at the first trimester, cortisol (µg/dl) at the first trimester, carrier of HFE gene mutation (no (ref.), yes for stratum 2), and gestational weight gain (kg). The significance of numbers in bold is *p*-value < 0.05. SGA (<10th percentile) or LGA (>90th percentile) versus appropriate-for-gestational-age (10 to 90th percentile) for gestational age- and sex-specified birthweight, length, or head circumference based on the INTERGROWTH-21st standards as reference. The diamonds represent OR and the whisker plots represent 95% CIs. n—number of cases infants (%); N—number of total infants.

The prevalence of SGA for birth weight, length, and HC was 11.1%, 11.9%, and 5.9%, respectively, and the prevalence of LGA for birth weight, length, and HC was 9.8%, 9.7%, and 21.5%, respectively. As shown in Figure 3, the multivariable analysis revealed a significantly increased risk of delivering an LGA baby (OR = 2.35; 95% CI 1.18–4.67; *p* = 0.015) and a non-significant risk of delivering an SGA baby (OR = 1.60; 95% CI 0.82–3.15; *p* = 0.169) in women from stratum 1 with a daily prenatal iron dose of 80 mg, compared to those who received 40 mg/d. 

No further significant differences were observed in the other parameters.

## 4. Discussion

To our knowledge, this is the first trial to examine the effectiveness of various doses of prenatal iron supplementation based on initial Hb levels in non-anemic women on the fetal growth parameters throughout the entire pregnancy up to delivery. We found that tailoring iron supplementation to the pregnant woman’s status achieves good results in fetal development. However, high prenatal doses of iron in each stratum increased the risk of having fetal and birth development below and above the “optimal” growth. In stratum 1, a daily prenatal dosage of 80 mg iron increased the risk of having both an SGA (<10th percentile) and LGA (>90th percentile) baby in terms of weight, not only at the end of gestation but also at birth. Regarding stratum 2, 40 mg per day of iron taken prenatally increased the risk of babies having an HC above the 90th percentile in the third trimester. 

Recent meta-analyses [22,45] have found a “U”-shaped risk for maternal iron status during pregnancy, suggesting that both iron deficiency and excess may negatively affect fetal growth and development. This would justify prescription of suitable prenatal iron supplementation tailored to the mother’s initial iron stores, aiming to avoid both adverse conditions. The current study reinforces this. Our results emphasize the importance of early universal screening for maternal iron status, along with personalizing the dose of iron supplementation appropriate to each woman’s needs, i.e., adapted to their initial Hb levels and monitoring iron parameters during pregnancy. This is crucial to avoid iron overload, especially in at-risk, iron-replete pregnant women, which would potentially lead to improved fetal growth. The findings are particularly important for clinical practice, as current global guidelines advocate prophylactic prenatal iron supplementation regardless of a woman’s iron status [9,10,11], and healthcare providers often pay more attention to anemia.

Supporting our results, extensive research from both RCTs and observational studies demonstrates that high-dose iron supplementation in non-anemic, iron-replete women can negatively impact fetal growth, based on birth anthropometric measurements. For instance, an RCT [23] assessing 727 Iranian non-anemic pregnant women found that iron supplementation with a dose of 50 mg/d of iron in women with Hb ≥ 13.2 g/dl in early pregnancy increases the risk of the baby being SGA at birth in comparison with those in the placebo group. Similarly, an observational cohort study of 1196 non-anemic pregnant women in South India revealed that those in the highest tertile (>39.2 mg/d) of supplemental iron intake exhibited an elevated risk of delivering LBW infants compared to those in the lowest tertile (≤36.6 mg/d) [24]. This raises concerns about the decision regarding the prescribed doses of prenatal iron supplements for already iron-replete pregnant women, as excess prenatal iron could increase the risks of fetal growth restriction.

In addition, a retrospective study by Zhiguo Wang et al. [46] reported that a high level of maternal plasma ferritin (>70 ng/mL) was associated with macrosomic newborns (birthweight > 4000 g). Similarly, a large cohort study carried out by Xiao-Guo Hua et al. [47] with a population of pregnant women in China reported that prenatal iron and folic acid (FA) supplementation (FA, 0.4 mg; iron, 30 mg or more per day) regardless of initial iron levels was positively linked to a higher risk of LGA or macrosomia, compared with the non-supplemented group.

An RCT also tested the effects of two different doses of antenatal daily iron supplements (containing 60 mg in the iron–folic acid group and 30 mg in the multiple micronutrient group) versus supplements of folic acid alone (400 µg) in a non-anemic rural pregnant population (initial Hb ≥ 11 g/dl) in China [48]. The authors reported a modest increase in birthweight and a non-significant 21% reduction in the risk of LBW (<2500 g) in the women who received a multiple micronutrient supplement containing a low iron dose, while supplements with high doses of iron–folic acid had no effect on birth outcomes in terms of weight. Likewise, in a US trial, low iron supplementation (30 mg/d) versus a placebo from 11 to 28 weeks of gestation in pregnant women who were not anemic at the beginning of pregnancy led to a notable increase in mean birth weight, of approximately 206 g, and significantly decreased the risk of LBW [20]. Taken together, these results and our data suggest that a prenatal iron administration of 30–40 mg/d (the iron dose commonly prescribed in Spain) for pregnant women with adequate iron stores may benefit fetal growth more than a high dose of iron.

At this point, it is noteworthy that, to date, there are no published randomized controlled trials in the latest research regarding prenatal iron supplements and focusing on fetal intrauterine growth parameters, hindering direct comparisons with the current results. To the authors’ knowledge, only one prospective observational cohort study has researched the association between total iron intake from the diet and supplements and fetal biometry, comprising 337 pregnant women at mid-pregnancy (around 28th weeks) in Republic of Korea. Using the data from the Mothers’ and Children’s Environmental Health (MOCEH) study, Hwang et al. [26] reported that 65% of the supplement users had a daily iron intake above the upper level (UL, 45 mg). In their study, despite an inadequate iron intake from food, the iron intake of supplement users was 76.9 ± 48.5 mg. The BD, AC, and FL of the babies of mothers in the third tertile of total iron intake (>17.04 mg), were significantly lower than the BD, AC, and FL of the babies of mothers with lower iron intake (11.49–17.04 mg/d). 

Supporting our results, various pathways have been postulated that demonstrate the possible pathophysiological mechanisms by which prenatal iron supplementation at high daily doses in non-anemic pregnant women with adequate iron reserves can negatively impact optimal fetal growth, particularly towards the end of gestation. It is understood that as pregnancy advances, the body’s ability to absorb iron progressively increases, with a notably significant rise during the third trimester, because iron is crucial for proper fetal growth [49]. However, an excess of plasma iron may result in non-transferrin-bound iron, a redox-active form of free iron that facilitates the creation of reactive oxygen species (ROS), which can be potentially toxic [50]. In fact, in a previous study by our group, higher circulating levels of iron, as measured by SF and/or transferrin saturation, were associated with increased lipid peroxidation (measured with the oxLDL/LDL ratio) in the general population [51]. In a recent study, prenatal iron supplementation of 60 mg/day in non-anemic pregnant women significantly increased oxidative stress, measured by the malondialdehyde/total antioxidant status ratio and levels of C-reactive protein, a marker for low-grade inflammation, throughout pregnancy [52]. Thus, the oxidative stress that stems from the accumulation of free iron may have both direct and indirect adverse effects on fetal growth. This can lead to ROS-associated lipid peroxidation and damage to the DNA of fetal cells, potentially resulting in conditions such as premature birth, intrauterine growth restriction, LBW and SGA offspring, among other complications [53]. Similarly, an abundance of circulating free iron can induce a potentially harmful inflammatory state, generating proinflammatory cytokines and activating immune cells, which may negatively influence fetal growth by disrupting placental functions, such as nutrient and oxygen transfer to the fetus, and causing tissue damage in the fetus, thus impacting normal fetal development [54,55,56]. It has also been hypothesized that maternal iron accumulation could raise blood viscosity, thereby compromising uteroplacental flow and, consequently, leading to suboptimal fetal growth [23]. As a result, pregnant women who already have sufficient iron and receive excessive daily iron supplementation might be at increased risk of iron overload and toxicity, thereby exposing the developing fetus to damage associated with the oxidative potential of circulating free iron, which is notably prevalent in the placenta [57].

Iron overload during pregnancy is especially linked with a heightened risk of certain pregnancy complications, such as hypertensive disorders [58] and gestational diabetes [8], which can impinge on intrauterine growth and birth outcomes. Indeed, it has been posited that an excessive intake of iron through the diet in pregnant women with ample iron reserves can heighten oxidative stress, which leads to insulin resistance and an insufficient compensatory maternal insulin response, resulting in fetal hyperglycemia and hyperinsulinemia [59]. This, in turn, can foster rapid fetal growth and excessive fat deposition, which are closely associated with fetal macrosomia [60]. Finally, it is crucial to consider that excess iron can interfere with the absorption of other essential micronutrients, such as zinc and copper, by competing for absorption transporters within the gastrointestinal tract. This competition can lead to micronutrient deficiencies, detrimentally affecting fetal growth and development by impeding critical cellular and enzymatic functions [61]. 

Similarly, maternal iron deficiency during pregnancy can adversely affect fetal growth and development due to diminished oxygen transport, as iron is a vital component of Hb [62]. Furthermore, iron is an essential cofactor for DNA synthesis and cell division, and therefore a deficiency could lead to delayed fetal growth [63]. Finally, iron deficiency can weaken the immune function, increasing susceptibility to infections and negatively impacting fetal growth trajectories [64].

Considering the negative influences of both iron deficiency and excess during pregnancy, it is imperative to maintain proper iron homeostasis during this period to achieve better outcomes for the mother and to ensure optimal fetal growth and development. Milman [65] suggested that it is preferable to use individual iron prophylaxis based on serum ferritin levels, which are indicative of the risk of iron deficiency, than general prophylaxis. Indeed, in an earlier analysis in the same cohort of the ECLIPSES study, it was found that adjusting prenatal iron supplementation in nonanemic women to their initial iron status proved to be an effective strategy in preventing both ID and iron excess in at-risk participants [6]. The results of our current study further support these recommendations.

The main strength of this study lies in it being the first to research the effect on fetal growth of iron supplementation doses adjusted to initial Hb levels, using a relatively large sample of non-anemic women and assessing fetal growth through fetal biometrics obtained with ultrasounds. It should be noted that excluding anemic women was important in this study, as our research strategy focuses on the primary prevention of anemia, not its treatment. In addition, this study has advantages over previous research as it was a triple-blind randomized clinical trial, which enhances its methodological rigor. We were able to assess the progression of the iron status and fetal growth by monitoring blood parameters and ultrasounds in all three trimesters of pregnancy. Moreover, it was found that the initial characteristics of the population studied did not differ significantly between the different iron supplement groups within each stratum. This indicates that the randomization process successfully balanced the treatment groups, which increases the reliability of the results. It is also important to recognize the inherent limitations of this trial. Firstly, the study was limited to a specific population from the northern Mediterranean coast of Spain, which might affect the extrapolation of the results to other geographical and cultural contexts. Secondly, it would be interesting to continue the research to elucidate the effects of iron supplementation on other parameters of fetal development, such as brain development, which is not addressed in this work. And finally, the low prevalence of iron deficiency in early pregnancy among our non-anemic participants limited the assessment of its impact on fetal growth.

## 5. Conclusions

Iron supplementation adjusted to initial Hb levels in in non-anemic pregnant woman seems to obtain good results in fetal development at any dose within each stratum. However, excessive doses of iron appear to negatively influence optimal fetal growth. Specifically, high prenatal doses of iron in each stratum increased the risk of fetal and birth development falling below or exceeding the “optimal” growth parameters. These findings highlight the necessity to implement individualized iron supplementation protocols that consider the Hb levels of non-anemic pregnant women, with the goal of optimizing outcomes related to fetal growth. Therefore, we recommend carrying out future trials that allow for more in-depth research into these issues, as well as determining the optimal dose of iron supplementation that maximizes the benefits for both the mother and the neonate, while minimizing the associated risks.

## Figures and Tables

**Table 1:** Baseline characteristics of the study population according to iron supplementation groups by stratum.

	Iron Supplementation Group					
	Stratum 1			Stratum 2		
	40 mg/d	80 mg/d	*p* Value	20 mg/d	40 mg/d	*p* Value
Characteristics	N = 261	N = 268		N = 130	N = 132	
Age (years), mean ± SD	30.7 ± 4.3	30.1 ± 5.3	0.217	30.2 ± 4.9	30.1 ± 5.6	0.812
BMI (kg/m^2^), mean ± SD	24.6 ± 4.1	24.7 ± 4.1	0.757	25.7 ± 5.3	25.8 ± 4.8	0.856
BMI categories, n (%)						
18.5–24.9 (normal weight)	198 (64)	162 (60)		74 (57)	67 (51)	
25.0–29.9 (overweight)	61 (24)	74 (28)	0.530	31 (24)	42 (32)	0.355
≥30 (obese)	32 (12)	32 (12)		25 (19)	23 (17)	
GWG (kg), mean ± SD	10.5 ± 3.5	10.3 ± 3.7	0.448	10.0 ± 3.9	10.5 ± 3.9	0.322
Educational level, n (%)						
Low (primary or less)	88 (34)	87 (32)		46 (35)	40 (30)	
medium (secondary)	103 (39)	104 (39)	0.882	47 (36)	49 (37)	0.639
High (university or more)	70 (27)	77 (29)		37 (29)	43 (33)	
Family SES, n (%)						
Low	39 (15)	47 (18)		24 (18)	18 (14)	
Medium	174 (67)	180 (67)	0.521	87 (67)	89 (67)	0.431
High	48 (18)	41 (15)		19 (15)	25 (19)	
Cigarette smoking, n (%)						
No	222 (85)	219 (82)	0.302	104 (80)	105 (80)	0.927
Yes	39 (15)	49 (18)		26 (20)	27 (20)	
Alcohol consumption, n (%)						
No	213 (87)	211 (87)	0.934	106 (86)	107 (89)	0.479
Yes	32 (13)	31 (13)		17 (14)	13 (11)	
Physical activity (METs—min/week), n (%)						
Sedentary/low (<600)	49 (19)	54 (20)		28 (22)	39 (30)	
Moderate (≥600–1200)	41 (16)	39 (15)	0.884	20 (15)	14 (11)	0.234
High (≥1200)	171 (66)	175 (65)		82 (63)	79 (60)	
Energy intake (kcal), mean ± SD	2095 ± 460	2123 ± 477	0.510	2121 ± 544	2165 ± 528	0.522
Dietary iron intake (mg), mean ± SD	7.8 ± 2.7	7.7 ± 2.4	0.628	7.7 ± 2.8	7.6 ± 2.2	0.752
Dietary vitamin C intake (mg), mean ± SD	76.5 ± 33.8	78.3 ± 35.6	0.582	77.5 ± 38.5	75.4 ± 37.2	0.658
Fiber intake (g), mean ± SD	12.5 ± 4.6	12.7 ± 4.3	0.623	12.5 ± 5.1	12.2 ± 4.1	0.648
Parity, n (%)						
Primiparous	94 (36)	105 (39)	0.473	57 (44)	59 (45)	0.890
Multiparous	166 (64)	163 (61)		73 (56)	73 (55)	
**Iron status characteristics**						
Hb (g/L), mean ± SD	123.4 ± 4.7	123.3 ± 5.3	0.690	136.6 ± 4.4	135.7 ± 4.6	0.101
SF (µg/L), mean ± SD *	32.1 ± 2.0	32.8 ± 2.1	0.740	34.8 ± 1.9	34.8 ± 1.9	0.965
Iron deficiency, n (%) **	37 (14)	38 (14)	0.999	16 (12)	19 (14)	0.620
Carrier of HFE gene mutation, n (%)	62 (30)	74 (36)	0.164	32 (30)	40 (38)	0.192
H63D mutation, n (%)	51 (24)	63 (31)	0.150	29 (27)	34 (32)	0.377
S65C/C282Y mutation, n (%)	13 (6)	14 (7)	0.802	3 (3)	8 (8)	0.110
Cortisol (µg/dl), mean ± SD	17.6 ± 4.7	18.4 ± 4.6	0.064	18.6 ± 5.5	18.3 ± 5.2	0.693

Values are mean ± SD or number (%). Abbreviations: BMI—body mass index; GWG—gestational weight gain; SES—socioeconomic status; METs—metabolic equivalent of task; Hb—hemoglobin; SF—serum ferritin; HFE—hemochromatosis. *p*-value for the differences across supplementation dose as derived from independent samples Student’s *T*-test or chi-square test. The reference group was 40 mg/d dose group for stratum 1 and 20 mg/d dose group for stratum 2. * Geometric means of log-transformed. ** Defined as a serum ferritin concentration SF < 15 µg/L according to WHO definition.

**Table 2:** Effect of the intervention with iron supplementation throughout pregnancy on stratum 1 (40 or 80 mg/d) and stratum 2 (40 or 20 mg/d) regarding fetal growth characteristics (outcomes) *.

	Fetal Growth Parameters									
Period of Fetal Growth Measurement and Iron Supplementation	Estimated Fetal Weight (g)		Femur Length (mm)		Head Circumference (mm)		Biparietal Diameter (mm)		Abdominal Circumference (mm)	
	β (95% CI)	*p* Value	β (95% CI)	*p* Value	β (95% CI)	*p* Value	β (95% CI)	*p* Value	β (95% CI)	*p* Value
Second trimester										
Stratum 1										
Iron supplementation **: (40 mg/d (ref.) vs. 80 mg/d)	1.43 (−4.40, 7.26)	0.630	−0.05 (−0.40, 0.30)	0.780	0.26 (−1.14, 1.67)	0.711	−0.12 (−0.57, 0.33)	0.588	1.26 (−0.03, 2.54)	0.056
Stratum 2										
Iron supplementation **: (20 mg/d (ref.) vs. 40 mg/d)	1.54 (−7.44, 10.53)	0.735	0.28 (−0.22, 0.79)	0.267	0.03 (−1.52, 1.59)	0.965	0.07 (−0.51, 0.65)	0.813	−0.58 (−2.55, 1.38)	0.559
Third trimester										
Stratum 1										
Iron supplementation †: (40 mg/d (ref.) vs. 80 mg/d)	−9.77 (−41.11, 21.57)	0.540	−0.30 (−0.75, 0.14)	0.183	−0.15 (−1.76, 1.46)	0.855	−0.32 (−0.78, 0.51)	0.184	−0.30 (−2.32, 1.72)	0.767
Stratum 2										
Iron supplementation †: (20 mg/d (ref.) vs. 40 mg/d)	13.51 (−27.41, 54.47)	0.515	0.03 (−0.57, 0.62)	0.929	−0.05 (−2.42, 2.31)	0.963	0.13 (−0.66, 0.92)	0.744	2.36 (−0.47, 5.19)	0.103
	**Birth parameters**									
	**Birthweight (g)**			**Length (cm)**				**Head circumference (mm)**		
At birth	**β (95% CI)**		***p* value**	**β (95% CI)**			***p* value**	**β (95% CI)**		***p* value**
Stratum 1										
Iron supplementation †: (40 mg/d (ref.) vs. 80 mg/d)	−14.74 (−90.37, 60.89)		0.702	−0.02 (−0.41, 0.38)			0.939	−0.21 (−0.53, 0.10)		0.180
Stratum 2										
Iron supplementation †: (20 mg/d (ref.) vs. 40 mg/d)	18.53 (−98.35, 135.52)		0.755	−0.24 (−0.82, 0.34)			0.414	−0.06 (−0.56, 0.45)		0.823

Results are from multivariable linear regression analyses, which were run separately for each fetal growth parameter and for each period (second trimester, third trimester, at birth). Values are regression β coefficient (β) and 95% confidence intervals (CIs) and reflect the difference in growth for each fetal parameter compared to iron supplementation group of 40 mg/d for stratum 1 and 20 mg/d for stratum 2. * All fetal growth parameter values were corrected for gestational age (weeks) at the time of ultrasound measurement or for gestational age (weeks) at birth using residual method. ** Model 1: adjusted for sex of baby, maternal age (years), maternal early-pregnancy BMI categories (normal weight (ref.), overweight, obese), family SES (low (ref.), medium, high), smoking during pregnancy (no (ref.), yes), fiber intake at the first trimester, log-serum ferritin (µg/L) at the first trimester, cortisol (µg/dl) at the first trimester, and carrier of HFE gene mutation (no (ref.), yes for stratum 2). † Model 2: model 1 and additionally adjusted for gestational weight gain (kg). Ref—reference.

## Data Availability

The data presented in this study are available on request from the corresponding author. The data are not publicly available due to subject confidentiality.

## Supplementary Materials

The following supporting information can be downloaded at: https://www.mdpi.com/article/10.3390/nu16030437/s1, Table S1: Comparison of fetal growth parameters during pregnancy and birth parameters according to iron supplementation groups; Figure S1: Multivariable-adjusted ORs (95%(CIs) of low (<25th percentile) and high (>85th percentile) fetal growth in the second and third trimester pregnancy associated with maternal iron supplementation throughout pregnancy on stratum 1 (80 or 40 mg/d) and stratum 2 (40 or 20 mg/d).
