# Supplementary material for: Effect of Prenatal Iron Supplementation Adapted to Hemoglobin Levels in Early Pregnancy on Fetal and Neonatal Growth—ECLIPSES Study
Source: Nutrients. 2024 Feb 1;16(3):437. doi: 10.3390/nu16030437 (PMC10857398; doi:10.3390/nu16030437)
Supplement: Supplementary file 1 [file nutrients-16-00437-s001.zip › nutrients-2795663-supplementary.pdf]

Effect of prenatal iron supplementation adapted to hemoglobin levels in early pregnancy on the fetal and neonatal growth. ECLIPSES Study

Table S1. Comparison of fetal growth parameters during pregnancy and birth parameters according to iron supplementation groups.

Parameters	Iron supplementation group							
	Stratum 1				Stratum 2			
		40 mg/d	80 mg/d	*p* value		20 mg/d	40 mg/d	*p* value
	N	Mean ± SD	Mean ± SD		N	Mean ± SD	Mean ± SD	
Fetal growth parameters								
First trimester (12 weeks)								
Gestational age (weeks)	529	11.9 ± 0.6	12.0 ± 0.6	0.699	262	11.9 ± 0.5	12.0 ± 0.6	0.521
Crown–rump length (mm)	525	59.9 ± 8.4	59.7 ± 7.8	0.741	262	60.5 ± 8.0	59.8 ± 7.3	0.465
Biparietal diameter (mm)	269	20.4 ± 2.5	20.5 ± 2.3	0.800	142	20.3 ± 2.7	20.7 ± 2.8	0.415
Second trimester (20 weeks)								
Gestational age (weeks)	425	20.0 ± 0.8	20.0 ± 0.7	0.715	214	20.0 ± 0.7	20.0 ± 0.8	0.586
Estimated fetal weight (g)	425	363.5 ± 63.0	363.7 ± 55.7	0.972	214	364.7 ± 55.4	359.6 ± 58.4	0.514
Femur length (mm)	425	33.0 ± 2.8	33.0 ± 2.5	0.965	214	32.8 ± 2.7	33.3 ± 2.5	0.139
Head circumference (mm)	423	177.5 ± 10.4	178.1 ± 10.6	0.607	214	178.0 ± 9.9	178.9 ± 8.7	0.496
Biparietal diameter (mm)	423	48.3 ± 3.1	48.1 ± 3.4	0.673	214	48.0 ± 3.0	48.3 ± 2.8	0.487
Abdominal circumference (mm)	425	152.5 ± 10.8	153.9 ± 10.1	0.189	214	153.0 ± 10.1	153.0 ± 9.6	0.974
Third trimester (33 weeks)								
Gestational age (weeks)	473	33.4 ± 1.2	33.3 ± 1.2	0.367	232	33.4 ± 1.4	33.4 ± 1.6	0.964
Estimated fetal weight (g)	473	2277.8 ± 288.2	2239.6 ± 292.5	0.155	232	2275.9 ± 305.5	2271.5 ± 382.6	0.923
Femur length (mm)	473	64.8 ± 3.0	64.4 ± 3.2	0.142	232	64.4 ± 3.5	64.4 ± 3.1	0.981
Head circumference (mm)	472	304.6 ± 12.8	304.2 ± 13.4	0.715	232	304.3 ± 15.4	303.7 ± 13.4	0.740
Biparietal diameter (mm)	472	84.9 ± 3.5	84.5 ± 4.0	0.214	232	84.7 ± 4.8	84.8 ± 4.1	0.880
Abdominal circumference (mm)	472	295.5 ± 16.1	294.7 ± 17.4	0.607	232	295.1 ± 19.7	296.9 ± 15.2	0.437
Birth parameters								
Gestational age (weeks)	451	39.6 ± 1.6	39.6 ± 1.5	0.934	232	39.6 ± 1.8	39.5 ± 1.5	0.375
Birthweight (g)	451	3294.9 ± 458.8	3279.3 ± 448.3	0.742	232	3254.5 ± 502.2	3324.4 ± 515.2	0.297
Birthweight-for-gestational-age (z-score)*	451	0.06 ± 0.88	0.01 ± 1.00	0.531	232	-0.02 ± 1.12	0.09 ± 0.99	0.434
Length (cm)	361	49.3 ± 1.9	49.2 ± 2.3	0.709	187	49.3 ± 2.2	49.5 ± 2.3	0.588
Length-for-gestational-age (z-score)*	361	-0.04 ± 0.96	-0.06 ± 1.14	0.875	187	-0.04 ± 1.13	-0.02 ± 1.16	0.922
Head circumference (cm)	281	34.6 ± 1.4	34.4 ± 1.6	0.170	162	34.5 ± 1.4	34.8 ± 1.9	0.349
Head circumference -for-gestational-age (z-score)*	281	0.59 ± 1.07	0.41 ± 1.11	0.170	162	0.54 ± 1.10	0.63 ± 1.15	0.622
Female sex, n (%)	496	138 (56)	121 (49)	0.105	254	61 (48)	61 (48)	0.904

Values are expressed as a mean ± SD (standard deviation). P value for the differences across supplementation dose as derived from independent samples Student's T-test. *z-scores according to the INTERGROWTH-21st standards.

Stratum 1

Second trimester	Iron supplementation 40 mg/d n(%)/N	Iron supplementation 80 mg/d n(%)/N	Iron supplementation 40 mg/d (reference) vs. 80 mg/d aOR (95% CI)*	*p* value
**Estimated fetal weight (g)**				
Low EFW (<25th percentile)	58(32.0)/181	45(25.7)/175	0.74 (0.46, 1.21)	0.232
High EFW (>85th percentile)	28(18.5)/154	33(20.2)/163	1.05 (0.58, 1.91)	0.868
**Femur length (mm)**				
Low FL (<25th percentile)	40(21.3)/188	40(21.7)/184	1.11 (0.66, 1.89)	0.687
High FL (>85th percentile)	22(12.9)/170	24(14.3)/168	1.25 (0.64, 2.46)	0.512
**Head circumference (mm)**				
Low HC (<25th percentile)	42(22.5)/183	47(26.6)/177	1.20 (0.72, 1.99)	0.483
High HC (>85th percentile)	24(14.3)/168	29(18.2)/159	1.34 (0.71, 2.52)	0.362
**Biparietal diameter (mm)**				
Low BD (<25th percentile)	43(23.5)/183	54(31.8)/170	1.59 (0.96, 2.63)	0.070
High BD (>85th percentile)	27(16.2)/167	36(23.7)/152	1.41 (0.77, 2.58)	0.266
**Abdominal circumference (mm)**				
Low CA (<25th percentile)	60(32.6)/184	42(24.0)/175	0.68 (0.41, 1.11)	0.121
High CA (>85th percentile)	26(17.3)/150	33(29.9)/166	1.23 (0.68, 2.23)	0.499
**Third trimester**				
**Estimated fetal weight (g)**				
Low EFW (<25th percentile)	47(23.7)/198	69(33.3)/207	**1.74 (1.09, 2.78)**	**0.011**
High EFW (>85th percentile)	28(15.1)/185	32(19.4)/165	1.49 (0.81, 2.72)	0.196
**Femur length (mm)**				
Low FL (<25th percentile)	54(27.1)/199	58(29.3)/198	1.20 (0.75, 1.92)	0.437
High FL (>85th percentile)	34(19.0)/179	37(20.9)/177	1.13 (0.66, 1.94)	0.655
**Head circumference (mm)**				
Low BD (<25th percentile)	47(24.2)/194	59(28.4)/208	1.35 (0.85, 2.15)	0.199
High BD (>85th percentile)	38(20.5)/185	27(15.3)/176	1.12 (0.64, 1.96)	0.693
**Biparietal diameter (mm)**				
Low HC (<25th percentile)	51(25.6)/199	61(30.8)/198	1.19 (0.75, 1.91)	0.459
High HC (>85th percentile)	33(18.2)/181	37 21.3)/174	0.65 (0.36, 1.17)	0.150
**Abdominal circumference (mm)**				
Low CA (<25th percentile)	59(29.3)/ 201	62(30.8)/201	1.09 (0.69, 1.72)	0.706
High CA (>85th percentile)	31(17.9)/173	34(29.7)/173	1.15 (0.66, 2.03)	0.619

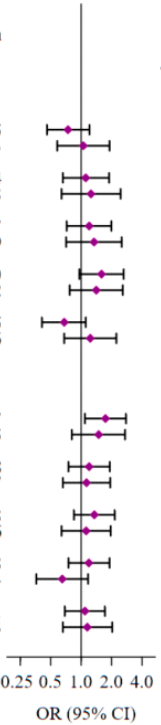

Stratum 2

Second trimester	Iron supplementation 20 mg/d n(%)/N	Iron supplementation 40 mg/d n(%)/N	Iron supplementation 20 mg/d (reference) vs. 40 mg/d aOR (95% CI)*	*p* value
**Estimated fetal weight (g)**				
Low EFW (<25th percentile)	26(27.1)/96	25(26.9)/93	0.92 (0.46, 1.85)	0.833
High EFW (>85th percentile)	12(14.6)/82	12(15.0)/80	1.01 (0.38, 2.67)	0.980
**Femur length (mm)**				
Low FL (<25th percentile)	23(23.7)/97	18(20.0)/90	0.95 (0.45, 1.99)	0.894
High FL (>85th percentile)	11(13.0)/85	15(17.2)/87	1.90 (0.70, 5.18)	0.204
**Head circumference (mm)**				
Low HC (<25th percentile)	31(33.3)/93	19(20.4)/93	0.59 (0.29, 1.21)	0.151
High HC (>85th percentile)	15(19.5)/77	12(14.0)/86	0.75 (0.30, 1.87)	0.573
**Biparietal diameter (mm)**				
Low BD (<25th percentile)	22(22.9)/96	22(24.2)/91	1.20 (0.58, 2.48)	0.621
High BD (>85th percentile)	12(14.0)/86	14(18.9)/83	1.27 (0.50, 3.22)	0.609
**Abdominal circumference (mm)**				
Low CA (<25th percentile)	23(25.8)/89	30(31.6)/95	1.47 (0.75, 2.89)	0.262
High CA (>85th percentile)	19(22.3)/85	10(13.3)/75	0.66 (0.27, 1.67)	0.380
**Third trimester**				
**Estimated fetal weight (g)**				
Low EFW (<25th percentile)	30(30.1)/97	28(28.0)/100	0.78 (0.48, 1.52)	0.470
High EFW (>85th percentile)	19(22.1)/86	16(18.2)/88	0.97 (0.43, 2.22)	0.953
**Femur length (mm)**				
Low FL (<25th percentile)	30(30.3)/99	26(25.2)/103	0.80 (0.41, 1.58)	0.529
High FL (>85th percentile)	17(29.8)/86	13(14.4)/90	0.68 (0.28, 1.63)	0.389
**Head circumference (mm)**				
Low HC (<25th percentile)	27(25.5)/106	32(32.6)/98	1.25 (0.64, 2.44)	0.507
High HC (>85th percentile)	10(11.2)/89	18(21.4)/84	**2.57 (1.05, 6.30)**	**0.039**
**Biparietal diameter (mm)**				
Low BD (<25th percentile)	32(32.0)/100	25(25.5)/98	0.63 (0.32, 1.23)	0.179
High BD (>85th percentile)	16(19.1)/84	18(19.8)/91	1.01 (0.45, 2.23)	0.988
**Abdominal circumference (mm)**				
Low CA (<25th percentile)	29(29.0)/100	22(22.2)/99	0.54 (0.26, 1.11)	0.100
High CA (>85th percentile)	17(18.1)/94	16(18.4)/87	1.08 (0.47, 2.45)	0.587

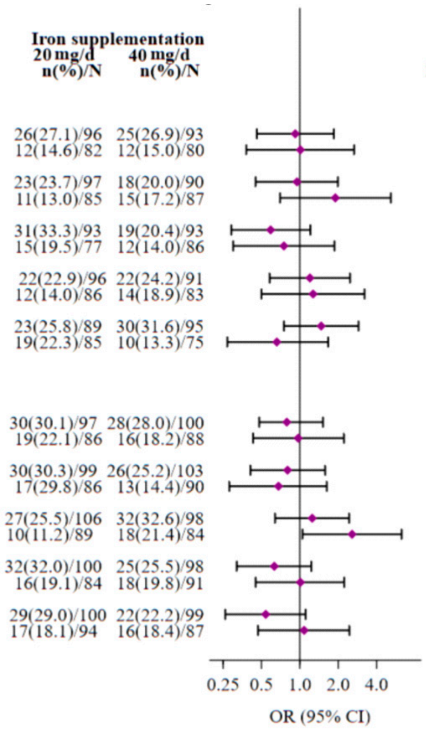

Figure S1. Multivariable-adjusted ORs (95%(CIs) of low (<25th percentile) and high (>85th percentile) fetal growth in the second and third trimester pregnancy associated with maternal iron supplementation throughout pregnancy on stratum 1 (80 or 40 mg/d) and stratum 2 (40 or 20 mg/d).

The reference group was 40 mg/d dose group for stratum 1 and 20 mg/d dose group for stratum 2. Abbreviations: EFW, estimated fetal weight; FL, femur length; HC, head circumference; BD, biparietal diameter; AC, abdominal circumference. All fetal growth parameter values were corrected for gestational age (weeks) at the time of ultrasound measurement using residual method. *Models were adjusted for sex of baby, maternal age (years), maternal early-pregnancy BMI categories (normal weight (ref.), overweight, obesity), family SES (low(ref.), medium, high), smoking during pregnancy (no (ref.), yes), fiber intake at the first trimester, log-serum ferritin (µg/L) at the first trimester, cortisol (µg/dl) at the first trimester, carrier of HFE gene mutation (no (ref.), yes for stratum 2), and gestational weight gain (kg, only for third trimester analysis). The significance of numbers in bold is p-value <0.05. Low (<25th percentile) or high (>85th percentile) fetal growth versus normal fetal growth (25th to 85th percentile) for gestational age-adjusted EFW, FL, HC, BD or AC based on distribution percentile from the study population as reference. The diamonds represent OR and the whisker plots represent 95% CIs. n, number of cases infants (%); N, number of total infants.
